# Spontaneous Regression of Hepatocellular Carcinoma and Review of Reports in the Published English Literature

**DOI:** 10.1155/2019/9756758

**Published:** 2019-03-31

**Authors:** Moaz B. Y. Chohan, Nick Taylor, Carla Coffin, Kelly W. Burak, Oliver F. Bathe

**Affiliations:** ^1^Departments of Surgery, University of Calgary, Calgary, AB, Canada; ^2^Departments of Medicine, University of Calgary, Calgary, AB, Canada; ^3^Departments of Oncology, University of Calgary, Calgary, AB, Canada

## Abstract

**Background:**

Spontaneous regression of hepatocellular carcinoma (HCC) is a rare event, although it has been described by numerous groups. The long-term fate of individuals experiencing an SR is not well described, and the underlying mechanism(s) of SR are unknown. *Case Presentation*: A 79-year-old Asian female with metastatic HCC taking only valsartan for hypertension had a marked reduction in tumor dimension in the primary tumor and the pulmonary metastases. Serum alpha-fetoprotein (AFP) decreased from 17,833 *μ*g/L to 26 *μ*g/L. Her disease progressed after 71 months, and she died shortly after. In a review of 66 patients with SR reported in the English literature, median survival was 83 months. Median survival in 37 cases that underwent resection after SR was 108 months.

**Conclusions:**

The case and a review of the literature illustrate that SR is often durable and associated with an excellent prognosis. Understanding the underlying mechanism of SR may point to novel therapeutic strategies.

## 1. Background

Hepatocellular carcinoma is the third most common cause of cancer death worldwide. Resection, liver transplantation, and ablation are the only potentially curative treatments. However, HCC is frequently accompanied by underlying liver disease, and multifocal disease additionally limits surgical options. Currently, the only approved systemic therapies for HCC are sorafenib and regorafenib, which are only beneficial with good liver function and which infrequently induces a measurable response [1, 2]. Therefore, there is a need to identify novel treatment strategies that can be delivered to patients with poor liver function and poor performance status.

Spontaneous regression of tumors is rare, estimated to occur in 1 in 60,000–100,000 patients [3]. SR of hepatocellular carcinoma is estimated to occur in 1 in 140,000 cases [4]. The literature is replete with case reports. However, little is known about the mechanism of SR. Moreover, case reports shed little light on the durability of SR. Understanding the mechanism of SR may provide new insights into treatment options, particularly if the result is a durable survival. We present a case of SR in an elderly patient taking valsartan, who would not be a good candidate for currently available treatments. In addition, we have reviewed the literature for evidence of a putative mechanism for SR as well as data on the long-term outcomes associated with this rare entity. Finally, we speculate on a potential role for angiotensin blockade in this case of SR of HCC.

## 2. Case Presentation

A 79-year-old Chinese female with chronic hepatitis C was referred for assessment of a liver mass. The patient was asymptomatic with no signs of decompensated liver disease. Her only comorbidity was well-controlled essential hypertension, for which she took valsartan 80 mg once daily. It was not clear from the history when she had started taking valsartan. She denied taking any specific herbal remedies. She was a nonsmoker and had no history of excess alcohol ingestion.

**Figure 1:**
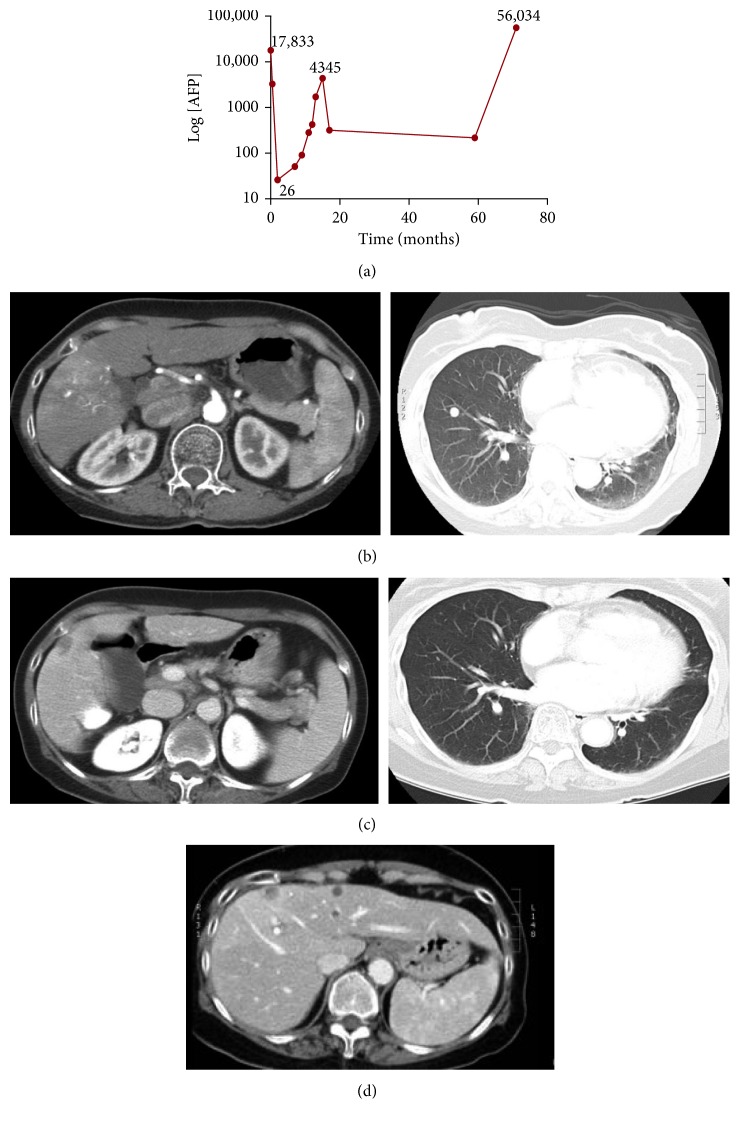
Spontaneous regression of HCC. (a) Serial changes in AFP levels (expressed as Log 10) over 18 months of follow-up and the time points of CT-scans. (b) A 4 × 4 cm liver lesion and bilateral lung lesions. (b) CT scan two months after diagnosis: the liver lesion has diminished in size (2.5 × 1.5 cm), and the lung lesions are barely visible. (c) CT scan at 17 months. The *l* liver lesion continues to be stable (now measuring 1.2 × 1.1 cm). (d) Recurrent liver metastases.

Her physical examination on admission was unremarkable. Her liver edge was nontender and palpable 2 cm below the right costal margin. There were no stigmata of chronic liver disease. Liver enzymes and function tests were in the normal range: (bilirubin 8 *μ*mol/L (normal 0–20), albumin 37 g/L (normal 33–48), INR 1.0 (normal 0.9–1.1), and creatinine 55 *μ*mol/L (normal 45–100)). The Child-Turcotte-Pugh Score was 5, and the raw MELD score was −1. Platelet count was depressed at 100 × 10^9^/L (normal 150–450). Serum alpha-fetoprotein (AFP) was extremely elevated at 17,833 *μ*g/L (normal 0–10; Figure 1(a)). Other investigations for chronic liver disease (i.e., hepatitis B virus, autoimmune, and metabolic etiologies) were negative. A triphasic computed tomography (CT) scan revealed a 4.0 × 4.0 cm mass in the right hepatic lobe and three nodules in the lower lung lobes (Figure 1(b)).

The patient was given a clinical diagnosis of hepatocellular carcinoma (HCC) with probable pulmonary metastases. She declined a liver biopsy. The disseminated state of her disease precluded ablative treatments and, because of her advanced age and frailty, she was not considered a candidate for chemotherapy.

At two months, the patient did not experience a decline in her condition, and a follow-up CT was done. The lung lesions had completely disappeared, and there was a significant decrease in the size of the primary liver lesion (Figure 1(c)). Serum AFP levels had fallen to almost within the normal range at 26 *µ*g/L (Figure 1(a)). During the subsequent 18 months, the patient remained clinically stable with no evidence of decompensated liver disease. There was a temporary rise in AFP, peaking at 4345 *μ*g/L, and then AFP decreased again to 319 *μ*g/L (Figure 1(a)). At 5, 8, 13, and 17 months, the liver lesion continued to shrink. Follow-up was deintensified. At 59 months, the AFP was 217. Then, at 71 months, she presented to the emergency department with ascites. A CT demonstrated a marked adverse change in the appearance of the liver: there were numerous solid and enhancing hepatic deposits (Figure 1(d)), as well as a large conglomerate solid mass measuring 10.6 × 6.3 × 8.0 cm in the right hepatic lobe. In addition, there were innumerable pulmonary nodules bilaterally. AFP was 56,034 *μ*g/L (Figure 1(a)). She died two weeks later. The patient's clinical trajectory is summarized in Supplementary Table S1.

## 3. Discussion

We report a case of SR of HCC, which ultimately led to progression following a long period of stability in a patient who was otherwise unable to receive conventional treatments due to frailty. The case raised a number of questions, including whether there are any specific clinical features of patients who have experienced an SR; what the underlying mechanism is; whether the angiotensin receptor blocker valsartan could have contributed to the SR; and how durable an SR is in reported cases. To address these questions, we performed a review with a literature search across five databases; EMBASE, PubMed, Medline, Cochrane Library, and CINAHL. The MeSH keywords employed for the search were a hepatocellular carcinoma, spontaneous regression, cancer regression, and outcome. The search included the following dates of publication: CINAHL, 1962 to present; EMBASE, 1974 to present; Cochrane Library of Systematic Reviews, 1999–February 2017; PubMed, no data date limits available; and MEDLINE, 1946–February 2017. Papers were excluded if any treatment directed at the HCC was administered before documented regression. We analyzed all case reports of SR of HCC in the English literature, which included 89 papers reporting 106 cases. The clinical characteristics of SR and the related long-term outcomes were extrapolated.

### 3.1. Characteristics of Cases of Spontaneous Regression of HCC

The clinical features of 106 cases were studied in detail and are summarized in Supplementary Table S2. These included 54 complete responses (50.9%) and 46 partial responses. There were 6 cases where the degree of response was not reported. In five cases, there was an initial (partial) SR, then regrowth (progression), followed by another regression. Mean tumor size was 5.7 cm (range 1.5–13.0 cm). AFP levels were on average 26,377 ng/mL (range 4–452,100). In 85 cases (80.2%), all diseases were limited to the liver. In the remaining 21 cases, there were 13 cases of lung metastases, six bone metastases, and two were widely disseminated. The most common etiologies of HCC were hepatitis B (15.1%), hepatitis C (38.7%), and alcohol (11.3%). Cirrhosis was reported in only 32 cases (30.2%).

### 3.2. Putative Mechanisms Associated with SR

In cases reviewed, a number of putative mechanisms underlying SR of HCC have been proposed (Table 1). However, the evidence for each of these mechanisms is based on association and speculation, and no convincing data shed light on the true pathogenesis. We found 19 cases where resection was performed following SR and where there was sufficient description of the pathology (Supplementary Table S3). The radiological and pathological features of these tumors generally suggested that the lesions regressed either due to a vascular accident or due to an immune-mediated response.

SR has been most commonly attributed to a robust immune response against tumor. Perhaps the best evidence for any role in tumor immunity is the association of SR with the withdrawal of immunosuppressants [10]. SR has been reported to be preceded by improved control of diabetes [7] and with resolution of hepatitis infection [11], which presumably is accompanied by improved immune function. Infections (especially viral infections) may stimulate an immune response against the tumor, either by stimulating a systemic inflammatory response or by inducing tumor immunity [12–14]. The abscopal effect has been reported in association with SR [15], and SR has been associated with blood transfusions [16]. In each of these cases, it is conceivable that an immune-mediated mechanism could have been at work. An immune-mediated mechanism for SR would be most plausible for cases where multiple tumors regressed coincidentally.

Another plausible mechanism identified in cases is a vascular compromise. Tumors such as HCC with high metabolic rates are susceptible to SR in conjunction with a sudden fall in hepatic blood flow including rapid growth [13, 17, 18], arterioportal shunting [8, 19], and portal vein thrombosis [20, 21]. Thickening of the vessel intima leading to thrombus formation may also be an initiating factor [9]. In one case, a patient underwent angiography which resulted in regression due to the formation of a thromboembolus in the feeding arteries [22]. Patients undergoing dialysis have also undergone SR, thought to be secondary to hypoxia and hypotension [23, 24]. One related mechanism of SR is impaired nutrient access. SR has been reported in association with tumor capsule formation [5, 25]. In the absence of intracapsular blood flow, it is possible that oxygen and other nutrients are not being delivered at sufficient levels to maintain tumor viability.

Herbal and complementary medicines have been associated with some cases of SR. Vide infra and a mixture of Aloe arborescens, as well as Phellinus linteus, were associated with cases of SR, but the underlying mechanisms were unknown [14, 15, 26]. According to Cheng and Tsai [27], the mono-oxygenases of Gentianae scabrae radix modulate liver microsomal cytochrome P450. Solamargine purified from Solanum incanum triggers gene expression of human TNF receptor I, which may lead to cell apoptosis [28]; Forsythiae fructus protects against CC14 hepatic injury by inhibition of lipid peroxidation in hepatic microsomes [29]. Antidepressants [30] and vitamin B supplementation [31] have been reported in association with SR. Finally, SR has been associated with androgen withdrawal [32].

### 3.3. The Potential Role of Angiotensin Receptor Blockade

As far as we are aware, there have not been other cases of SR associated with angiotensin receptor blockade. The fact that both primary and metastatic sites regressed suggests that some kind of systemic effect had occurred. Admittedly, it would be highly speculative of us to attribute our case of SR to angiotensin receptor blockade. On the other hand, there is some evidence that angiotensin receptor blockade has a potentially antineoplastic effect. The role of the renin-angiotensin system (RAS) in the homeostasis of sodium, water, and potassium is well understood. In addition to the circulating RAS, there are tissues containing a local RAS, including cancers. Central to this is the role of angiotensin II, the ligand for the angiotensin receptors (AT1 and AT2). Signalling through the AT2 receptor seems to inhibit cancer progression. Du et al. demonstrated that AT2 receptor overexpression regulates proliferation of hepatocellular carcinoma cells *in vitro* and *in vivo*, but the precise mechanism of that phenomenon is yet to be determined [33]. In bladder cancer, the AT2 receptor promotes apoptosis and inhibits angiogenesis [34]. In contrast, signalling through the AT1 receptor appears to promote tumor progression. Blockade of the AT1 receptor with angiotensin receptor blocker losartan inhibits mammary tumor development and progression to invasive carcinoma [6]. AT1 receptor blockers have also been shown to enhance the effects of bevacizumab-based chemotherapy in metastatic colorectal cancer patients [35]. In addition to signalling through AT1 and AT2, angiotensin II can be converted to Ang-(1–7), which is catalyzed by ACE2. Ang-(1–7) is the ligand for the Mas receptor, and its activation has proneoplastic effects in hepatocellular carcinoma [36] and other malignancies [37, 38]. Finally, Ye et al. [36] investigated the biological pathways involved in HCC pathogenesis through the ACE2 pathway. They found that there was a negative correlation between the mRNA levels of ACE2 and CD34. Patients with a higher level of ACE2 expression had a longer survival time than those with lower levels of ACE2 expression.

### 3.4. Long-Term Outcomes after Spontaneous Regression

**Figure 2:**
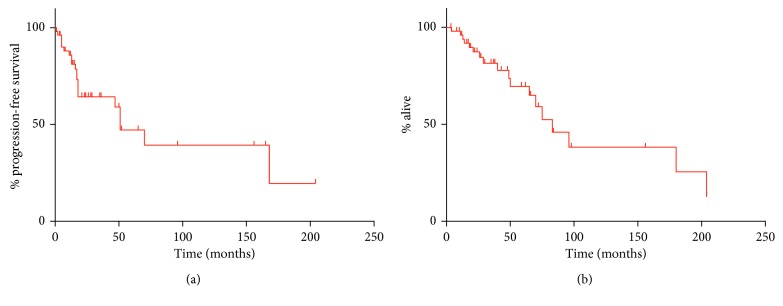
Long-term survival outcomes documented in cases of spontaneous regression of HCC that were treated nonsurgically, based on cases appearing in the English literature. (a) Kaplan–Meier curve, progression-free survival. (b) Kaplan–Meier curve, overall survival.

In the 66 cases of SR treated nonsurgically, median progression-free survival was 51 months (mo). Median overall survival of the 66 patients who had an SR but no additional treatment was 83 mo. Survival curves are depicted in Figure 2. In three of those cases, the cause of death was not related to the tumor. Of the 37 cases of SR treated with resection (after SR), follow-up was quite variable, ranging from 2–240 months. There were only three reported deaths, so median survival could not be estimated accurately.

## 4. Conclusions

SR is a rare phenomenon. Further studies are required to provide plausible data to identify a potential cause for SR of HCC, which is associated with excellent long-term survivals. To identify a potential mechanism of SR will be challenging. One potential means to do this is to routinely bank tissues (blood and tumor) of all cases of HCC before diagnosis and to obtain post-SR samples if they appear. Of course, due to the rare incidence of SR, obtaining such samples would represent a fortuitous event.

## Figures and Tables

**Table 1:** Putative mechanisms of spontaneous regression of HCC reported in the literature.

Putative mechanisms of SR	References
Inflammatory or immune mechanism: spontaneous, concomitant infection or inflammatory condition, abscopal effect, blood transfusion	[5]
Vascular compromise: rapid growth, vascular compression, drug effect, severe dehydration	[6]
Metabolic: weight loss, improved diabetic control	[7]
Miscellaneous or unknown drug effects: antidepressants, androgens/estrogens	[4]
Complementary and alternative medicine	[8]
Idiopathic	[9]

## Abbreviations

SR: Spontaneous regression
HCC: Hepatocellular carcinoma
AFP: Alpha-fetoprotein
INR: International normalized ratio
MELD: Model of end-stage liver disease
TNF: Tumor necrosis factor
RAS: Renin-angiotensin system.

## Supplementary Materials

Supplementary Materials Table S1: patient's clinical history organized as a timeline. Table S2: characteristics of patients with spontaneous regression (SR) of HCC. Table S3: resected cases of HCC that regressed spontaneously, with radiographic and pathologic features that demonstrate vascular insufficiency and/or an inflammatory response.
