# Supplementary material for: Spontaneous Regression of Hepatocellular Carcinoma and Review of Reports in the Published English Literature
Source: Case Rep Med. 2019 Mar 31;2019:9756758. doi: 10.1155/2019/9756758 (PMC6462316; doi:10.1155/2019/9756758)
Supplement: Supplementary Materials — Table S1: patient's clinical history organized as a timeline. Table S2: characteristics of patients with spontaneous regression (SR) of HCC. Table S3: resected cases of HCC that regressed spontaneously, with radiographic and pathologic features that demonstrate vascular insufficiency and/or an inflammatory response. [file 9756758.f1.zip › table s1.docx]

**Table S1: Timeline of disease progression and regression.**

| Timeline | Finding | Outcome |
| --- | --- | --- |
| Baseline | LFT’s and liver enzymes normal, INR normal, Creatinine normal, platelets depressed, serum AFP increased, CT scan shows 4 x 4 tumor in liver and three nodules in the lower lung lobes | Clinical diagnosis of HCC with lung metastasis. Patient denied biopsy. |
| 2 months | CT scan shows decrease in liver tumor size and disappearance of lung lesions. Serum AFP was normal. | Clinically stable. |
| 5 months | CT scan shows decrease in liver lesion size. | Clinically stable. |
| 8 months | CT scan shows decrease in liver lesion size. | Clinically stable. |
| 13 months | CT scan shows decrease in liver lesion size. | Clinically stable. |
| 17 months | CT scan shows decrease in liver lesion size. | Clinically stable. |
| 20 months | Temporary increase in AFP, then back to normal. | Clinically stable. |
| 59 months | Serum AFP was 217. | Clinically stable, follow-up de-intensified. |
| 71 months | CT scan showed increase liver tumor and multiple pulmonary metastasis. AFP 56,034. | Patient presented with ascites, died two weeks later. |
