# Supplementary material for: Spontaneous Regression of Hepatocellular Carcinoma and Review of Reports in the Published English Literature
Source: Case Rep Med. 2019 Mar 31;2019:9756758. doi: 10.1155/2019/9756758 (PMC6462316; doi:10.1155/2019/9756758)
Supplement: Supplementary Materials — Table S1: patient's clinical history organized as a timeline. Table S2: characteristics of patients with spontaneous regression (SR) of HCC. Table S3: resected cases of HCC that regressed spontaneously, with radiographic and pathologic features that demonstrate vascular insufficiency and/or an inflammatory response. [file 9756758.f1.zip › table s2-characteristics of patients with spontaneous regression_CRIM_2680984.docx]

**Table S2: Details of cases published in literature, their demographics, disease and treatment details if available.**

| **Paper Citation** | **Sex** | **Age** | **Etiology of HCC** | **Cirrhosis** | **AFP (ng/mL)** | **Site of Disease** | **Site of SR** | **Partial or Complete** | **Follow up (months)** | **Medications and alternate therapies** |
| --- | --- | --- | --- | --- | --- | --- | --- | --- | --- | --- |
| 1.    Blondon et al. No. 1 [1] | M | 64 | Alcohol | Y | 915 | Liver | Liver | Partial | 36 |  |
| 2.    Blondon et al. No. 2[1] | F | 70 | Alcohol | Y | 4000 | Liver | Liver | Partial | 9 |  |
| 3.    Ohba et al. [2] | M | 76 | Hepatitis C | Y | 429998 | Liver, bone | Liver | Partial | 29 |  |
| 4.    Terasaki et al. [3] | F | 72 | Unreported |  | 7600 | Liver, peritoneum, spleen | Liver, peritoneum, spleen | Complete | 24 |  |
| 5.    Zimmerman et al. [4] | M | 56 | Cirrhosis - unknown cause | Y | 24000 | Liver | Liver | Unreported | 25 |  |
| 6.    Toyoda et al. [5] | M | 82 | Hepatitis C |  | 50000 | Lung, liver | Lung | Partial | 21 |  |
| 7.    Arakawa et al. [6] | F | 78 | Hepatitis B |  | 1041 | Liver | Liver | Complete | 30 | None |
| 8.    McDermott and Khettry [7] | F | 23 | Unreported |  |  | Liver | Liver | Complete | 60 | None |
| 9.    Imaoka et al. [8] | M | 65 | Chronic Hepatitis |  | 5670 | Liver | Liver | Complete | Unreported |  |
| 10. Nakajima et al. [9] | M | 80 | Unreported |  |  | Liver | Liver | Partial | 6 |  |
| 11. Lee et al. No 1 [10] | M | 44 | Hepatitis C | Unreported | 12200 | Liver | Liver | Partial | 13 |  |
| 12. Lee et al. No 2 [10] | M | 63 | Hepatitis B | Unreported | 1410 | Liver | Liver | Partial | 37 |  |
| 13. Rizell et al. [11] | M | 58 | Hepatitis C |  | 25300 | Liver | Liver | Partial | 20 | Sirolimus |
| 14. Li et al. [12] | M | 53 | Unreported |  | 25 | Liver | Liver | Complete | Unreported | None |
| 15. Ozeki et al. [13] | F | 69 | Unreported |  | 1050 | Liver | Liver | Complete | 12 | None |
| 16. Meza-Junco et al. [14] | F | 56 | Hepatitis C | Y | 195 | Liver | Liver | Complete | 25 |  |
| 17. Markovic et al. [15] | M | 62 | Cirrhosis - Hepatitis B | Y | 11 | Liver | Liver | Complete | 96 | None |
| 18. Storey et al. [16] | M | 52 | Alcohol |  | 39403 | Lung, liver | Lung, liver | Complete | 36 |  |
| 19. Alqutub et al. [17] | M | 65 | Unknown |  | 6500 | Liver | Liver | Complete | 24 |  |
| 20. Nakayama [18] | F | 92 | Unknown |  | 140 | Liver | Liver | Complete | Unreported | None |
| 21. Lin et al. [19] | M | 42 | Hepatitis B |  |  | Liver | Liver | Partial | 24 | Complete lifestyle change (diet, activity, vit B, religion) |
| 22. Matsuo et al. [20] | M | 72 | Hepatitis C |  | 1000 | Liver | Liver | Partial | 12 |  |
| 23. Randolph et. Al [21] | M | 56 | Hepatitis C |  | 2854 | Liver | Liver | Complete | 22 | None |
| 24. Chien et al. [22] | M | 65 | Hepatitis B 5 |  | 10000 | Liver | Liver | Complete 13 | 30 | Herbal medicines |
| 25. Heianna et al. [23] | F | 70 | Hepatitis C |  |  | Lung, liver | Lung, liver | Partial | 60 | Radiofrequency ablation |
| 26. Harimoto et al. [24] | M | 73 | Unreported |  | 32215 | Lung, liver | Lung | Complete | 13 |  |
| 27. Ayres et al. [25] | F | 63 | Unknown |  | 7390 | Liver | Liver | Partial | 12 | None |
| 28. Bastawrous et al. [26] | M | 63 | Hepatitis C |  |  | Liver | Liver | Partial | Unreported | None |
| 29. Izuishi et al. [27] | M | 50 | Unreported |  | 16 | Liver | Liver | Complete | 60 |  |
| 30. Gaffet et al. [28] | M | 63 | Unknown |  | 2690 | Liver | Liver | Partial | 17 |  |
| 31. van Halteren et al. [29] | F | 72 | Alcohol |  |  | Liver | Liver | Complete | 28 |  |
| 32. Kaczynski et al. [30] | M | 73 | Unknown |  |  | Liver | Liver | Complete | 39 | None |
| 33. Misawa et al. [31] | M | 62 | Cirrhosis - Hepatitis B | Y | 1400 | Liver | Liver | Complete | Unreported | None |
| 34. Uenishi et al. [32] | M | 65 | Unknown |  |  | Liver | Liver | Partial | 12 |  |
| 35. Nakai et al. [33] | M | 76 | Cirrhosis - Hepatitis C | Y |  | Liver | Liver | Complete | 24 |  |
| 36. Ohta et al. [34] | M | 74 | Unreported |  |  | Liver | Liver | Complete | 12 |  |
| 37. Kondo et al. No 1 [35] | M | 70 | Hepatitis C |  | 3360 | Liver | Liver | Partial | 63 |  |
| 38. Kondo et al. No 2 [35] | M | 75 | Hepatitis C 12 |  | 6750 | Lung, liver | Lung | Partial | 23 | None |
| 39. Kondo et al. No 3 [35] | M | 67 | Hepatitis C |  | 3385 | Liver | Liver | Partial | 2 |  |
| 40. Kondo et al. No 4 [35] | M | 67 | Hepatitis C |  | 601 | Lung, liver | Lung, liver | Complete | 48 |  |
| 41. Vardhana et. Al. [36] | M | Unreported | Unreported |  | 6705 | Liver | Liver | Unreported | 8 | None |
| 42. Sibartie et al. [37] | M | 76 | Alcohol |  | 1259 | Liver | Liver | Partial | Unreported |  |
| 43. Del Poggio et al. [38] | F | 77 | Hepatitis C |  | 3133 | Liver | Liver | Partial | 18 |  |
| 44. Iwasaki et al. [39] | F | 72 | Cirrhosis - unknown cause | Y | 743 | Liver | Liver | Partial | 20 | epirubicin and percutaneous etOH (posterior segment tumour) |
| 45. Lam et al. [40] | M | 50 | Hepatitis B |  |  | Lung, liver | Lung, liver | Complete | 156 | Chinese herbal medicine (vide infra) |
| 46. Tocci et al. [41] | M | 79 | Unknown | Y | 625 | Liver | Liver | Complete | 3 | IV hydrocortisone |
| 47. Takeda et al. [42] | M | 68 | Hepatitis C |  |  | Liver | Liver | Partial | 12 | Agaricus blazei Murill and tahibo tea |
| 48. Nishijima et al. [43] | F | 86 | Hepatitis C | Y |  | Liver | Liver | Partial | 4 | None |
| 49. Feo et al. [44] | F | 71 | Hepatitis C |  | 1750 | Liver | Liver | Partial | 18 | None |
| 50. Oquinena et al. No. 1 [45] | M | 54 | Hepatitis B 8 | Y | 465 | Liver | Liver | Complete | 22 | None |
| 51. Oquinena et al. No. 2 [45] | M | 61 | Cirrhosis - Alcohol | Y | 27353 | Liver | Liver | Complete | 18 | Transarterial embolization (TAE) |
| 52. Oquinena et al. No. 3 [45] | M | 60 | Unknown |  | 66 | Liver | Liver | Complete | 36 | Oxaliplatan and Gemcitabine |
| 53. Cheng and Tsai [46] | M | 74 | Unknown |  | 3500 | Liver | Liver | Complete | 72 | Variety of Chinese and Taiwanese herbs |
| 54. Sato et al. [47] | M | 78 | Chronic Hepatitis |  | 26200 | Liver, bone | Liver, bone | Complete 28 | 62 | Many various types (see paper for details) |
| 55. Abiru et al. No 1 [48] | M | 70 | Hepatitis C | Y | 226333 | Lung, liver, bone | Unreported | Unreported | 29 |  |
| 56. Abiru et al. No 2 [48] | F | 65 | Hepatitis C | Y | 166492 | Lung, liver, lymph node | Unreported | Unreported | 4 |  |
| 57. Abiru et al. No 3 [48] | M | 65 | Hepatitis C 21 | Y | 30092 | Lung, liver, bone | Unreported | Unreported | 11 |  |
| 58. Hong et al. [49] | M | 67 | Hepatitis B |  |  | Lung, liver | Unreported | Complete | 14 | None |
| 59. Nam et al. [50] | M | 65 | Hepatitis C | Unreported | 3100 | Liver, bone | Liver, bone | Partial | 15 | Phellinus linteus mushroom |
| 60. Stoelben et al. No. 1 [51] | M | 56 | Unknown |  | 4 | Liver | Liver | Complete | 24 | Surgical resection |
| 61. Stoelben et al. No. 2 [51] | M | 74 | Unknown |  | 3850 | Liver | Liver | Complete | 41 | Surgical resection |
| 62. Nouso et al. [52] | M | 85 | Hepatitis C |  | 1212 | Liver | Liver | Partial | 22 | Vitamin K |
| 63. Sanz et al. [53] | M | 66 | Hepatitis C |  | 4 | Liver | Liver | Complete | 16 | None |
| 64. Peddu et al. [54] | M | 57 | Cirrhosis - unknown cause | Y | 403 | Liver | Unreported | Complete | Unreported | None |
| 65. Ohtani et al. [55] | M | 69 | Hepatitis C |  |  | Liver | Liver | Partial | 37 | None |
| 66. Kato et al. No 1 [56] | M | 77 | Hepatitis C |  | 50000 | Lung, liver | Lung, liver | Complete | 12 | None |
| 67. Kato et al. No 2 [56] | M | 72 | Hepatitis C |  | 936 | Liver | Liver | Partial | 24 | None |
| 68. Hsu et al. [57] | M | 66 | Hepatitis C | Y | 4280 | Lung | Liver | Partial | 20 | None |
| 69. Yano et al. [58] | F | 71 | Hepatitis C |  | 1008 | Lung | Liver | Partial | 24 | Resection after sponstaneous regression and instant relapse. |
| 70. Takeura et al. No. 1 [59] | F | 69 | Hepatitis C | Y |  | Liver, bone | Liver, bone | Complete | 10 | None |
| 71. Takeura et al. No. 2 [59] | F | 84 | Unknown |  |  | Liver, peritoneum | Liver, peritoneum | Complete | 20 | None |
| 72. Arora and Madhusudhana [60] | M | 54 | Hepatitis C |  | 1027 | Liver | Liver | Complete | 48 | None |
| 73. Morimoto et al. [61] | M | 73 | Alcohol |  | 55 | Liver | Liver | Complete | 12 | None |
| 74. Gottfried et al. [62] | M | 65 | Alcohol | Y | 290 | Liver | Liver | Complete | 48 | None |
| 75. Saez-Royuela et al. [63] | M | 66 | Hepatitis B and Alcohol |  | 6400 | Liver | Liver | Complete | 31 | None |
| 76. Bruix et al. No. 1 [64] | M | 65 | Unknown |  |  | Liver | Liver | Partial | 35 | None |
| 77. Bruix et al. No. 2 [64] | M | 72 | Unknown |  | 4000 | Liver | Liver | Partial | 21 | None |
| 78. Bruix et al. No. 3 [64] | M | 64 | Unknown |  | 4800 | Liver | Liver | Partial | 44 | None |
| 79. Herrera et al. [65] | M | 76 | Unknown |  | 11240 | Liver | Liver | Complete | 14 | None |
| 80. McCaughan et al. No. 1 [66] | M | 28 | Unknown |  |  | Liver | Liver | Complete | 118 | None |
| 81. McCaughan et al. No. 2 [66] | M | 40 | Unknown |  |  | Liver | Liver | Complete 43 | 157 | None |
| 82. Takayasu et al. No. 1[67] | M | 38 | Hepatitis B |  |  | Liver | Liver | Partial | 2 | None |
| 83. Takayasu et al. No. 2 [67] | F | 58 | Cirrhosis | Y |  | Liver | Liver | Complete | 30 | None |
| 84. Chiesara et. Al. [68] | M | 65 | NASH (Non-Alcoholic Steatohepatitis) | Y |  | Liver | Liver | Partial regression | 24 | none |
| 85. Kumar et. Al. Pt. No. 1 [69] | M | 40 | Immunosuppression | No | 832 | Liver | Liver | Compelte Regression | 84 | None |
| 86. Kumar et. Al. Pt. No. 2 [69] | M | 74 | Immunosuppression | No | 96932 | Liver | Liver | Partial regression | 8 | None |
| 87. Park et. Al. [70] | M | 57 | Hepatitis B | Y | 17.3 | Liver | Liver | Partial regression | 60 | Surgical resection |
| 88. Yang et. Al. [71] | M | 56 | Hepatitis B | Unreported | 446 | Liver | Liver | Complete regression | 168 | None |
| 89. Emaculate et. Al. [72] | M | 53 | Hepatitis C | Y | 79 | Liver | Liver/Lung metastasis | Partial | 21 | None |
| 90. Mochizuki et. Al. [73] | M | 61 | Not mentioned | Y | 221000 | Liver | Liver | Partial | 18 | Radiation given for skull metastasis. |
| 91. Saito et. Al. [74] | M | 75 | Hepatitis C 34 | Unreported | 452100 | Liver | Liver | Partial | 84 | TACE |
| 92. Erturk et. Al. [75] | M | 69 | Hepatitis B | Unreported | 300 | Liver | Liver | Comeplete | 36 | None |
| 93. Yamamoto et. Al. [76] | M | 60 | unknown | Unknown |  | Liver | Liver | Comeplete | Not known. | None |
| 94. Wang et. Al. [77] | M | 50 | Hepatitis C | Unreported | 22592 | Liver | Liver | Partial | 24 | None |
| 95. Tsai et. Al. [78] | M | 74 | Hepatitis C | Y | 810.03 | Liver | Liver | Partial | 48 | None |
| 96. Okano et. Al. [79] | M | 73 | Hepatitis B | Unreported | 748 | Liver | Liver | Complete | 60 | None |
| 97. Okano et. Al. [80] | M | 77 | Not mentioned | Y | 1825 | Liver | Liver | Partial | 16 | TACE used for treating recurrent lesions |
| 98. Jozuka et. Al. [81] | M | 52 | Hepatitis C | No | 80.3 | Liver | Liver | Complete regression | 60 | None |
| 99. Matsuoka et Al. [82] | M | 67 | Non Alcoholic Steatohepatitis (NASH) | Y | 11 | Liver | Liver | Partial | 36 | Resection after sponstaneous regression. |
| 100. Tomino et. Al. [83] | M | 73 | Alcoholic liver damage | Unreported | 5.6 | Liver | Liver | Partial regression | 7 | Liver resection |
| 101. Suzuki et. Al. [84] | M | 65 | Alcoholic liver damage | Y | 494 | Liver | Liver | Complete regression | 70 | Hepatic arteriography before the event of spontaneous regression. |
| 102. Liai et. Al. [85] | M | 69 | Hepatitis C | Unreported | 3476.4 | Liver | Liver | Complete regression | 12 | Surgical resection |
| 103. Magalotli et. Al. Pt. No. 1 [86] | M | 66 | Alcoholic hepatitis | Y | 2500 | Liver | Liver | Complete regression | 48 | None |
| 104. Magalotli et. Al. Pt. No. 2 [86] | F | 75 | Hepatitis C, Hepatitis B 16 | Y | 37500 | Liver | Liver | Partial regression | 36 | None |
| 105. Sugamoto et. Al. [87] | F | 77 | Hepatitis C | Y |  | Liver | Liver | Complete regression 55 | Unknown | Surgical resection |
| 106. Ikeda et. Al. [88] | M | 75 | Hepatitis C 42 |  |  | Liver | Liver | Partial regression | 72 | TACE, Chemo |

**REFERENCES:**

1. Blondon, H., L. Fritsch, and D. Cherqui, *Two cases of spontaneous regression of multicentric hepatocellular carcinoma after intraperitoneal rupture: possible role of immune mechanisms.* Eur J Gastroenterol Hepatol, 2004. **16**(12): p. 1355-1359.

2. Ohba, K., et al., *Abscopal regression of hepatocellular carcinoma after radiotherapy for bone metastasis.* Gut, 1998. **43**(4): p. 575-577.

3. Terasaki, T., K. Hanazaki, and E. Shiohara, *Complete disappearance of recurrent hepatocellular carcinoma with peritoneal dissemination and splenic metastasis : A unique.* Journal of Gastroenterology and Hepatology, 2000(September 2000): p. 327-330.

4. Zimmermann, A., et al., *Hepatocellular carcinoma with an unusual medullary-like histology and signs of regression ("medullary-like hepatocellular carcinoma").* Dig Liver Dis, 2002. **34**(10): p. 748-753.

5. Toyoda, H., et al., *Hepatocellular carcinoma with spontaneous regression of multiple lung metastases.* 1999(May): p. 893-897.

6. Arakawa, Y., et al., *Hepatocellular carcinoma with spontaneous regression: Report of the rare case*. 2008. p. 1770-1772.

7. McDermott, W.V. and U. Khettry, *Clear cell carcinoma of the liver with spontaneous regression of metastases.* J Surg Oncol, 1994. **57**(3): p. 206-209.

8. Imaoka, S. and Y. Sasaki, *Necrosis of hepatocellular Carcinoma Caused by Spontaneously Arising Arterial Thrombus 1994.* Hepato-Gastroenterology, 1994. **41**: p. 359-362.

9. Nakajima, T., et al., *Recurrence of hepatocellular carcinoma with rapid growth after spontaneous regression.* World journal of gastroenterology : WJG, 2004. **10**(22): p. 3385-7.

10. Lee, H.S., et al., *Recurrent hepatocellular carcinoma after spontaneous regression.* Journal of Gastroenterology, 2000. **35**(7): p. 552-556.

11. Rizell, M., et al., *Impressive regression of primary liver cancer after treatment with sirolimus.* Acta Oncologica, 2005. **44**(5): p. 496-496.

12. Li, A.J., et al., *Spontaneous complete necrosis of hepatocellular carcinoma: A case report*. 2003. p. 152-154.

13. Ozeki, Y., N. Matsubara, and K.-i. Tateyama, *Spontaneous complete necrosis of hepatocellular carcinoma-Ozeki.pdf.* American Journal of Gastroenterology, 1996. **91**(2).

14. Meza-Junco, J. and A.J. Montano-Loza, *Spontaneous partial regression of hepatocellular carcinoma in a cirrhotic patient, Annals of Hepatology 2007.* Annals of Hepatology, 2007. **6**: p. 66-69.

15. Markovic, S. and V. Ferlan-Marolt, *Spontaneous regression of hepatocellular carcinoma.* American Journal of Gastroenterology, 1996. **91**(2): p. 392-393.

16. Storey, R.E., et al., *Spontaneous complete regression of hepatocellular carcinoma.* Med Oncol, 2011. **28**(4): p. 948-50.

17. Alqutub, A., D. Peck, and P. Marotta, *Spontaneous regression of HCC case report (2011).pdf.* German Medical Science, 2011. **9**.

18. Nakayama, S., *Spontaneous regression of hepatocellular carcinoma.* Indian J Gastroenterol, 2012. **31**(5): p. 267-70.

19. Lin, T.-J. and L.-Y. Liao, *Spontaneous Regression of Hepatocellular Carcinoma A Case Report & Literature Review, Hepatogastroenterology, 2004.* Hepato-Gastroenterology, 2004. **51**: p. 579-582.

20. Matsuo, R., et al., *Spontaneous regression of hepatocellular carcinoma - A case report.* Hepato-Gastroenterology, 2001(48): p. 1740-1742.

21. Randolph, A.C., E.M. Tharalson, and N. Gilani, *Spontaneous regression of hepatocellular carcinoma is possible and might have implications for future therapies.* Eur J Gastroenterol Hepatol, 2008. **20**(8): p. 804-809.

22. Chien, R.-N., T.J. Chen, and Y.-F. Liaw, *Spontaneous Regression of Hepatocellular Carcinoma,Chien RN, Chen TJ, Liaw YF, American Journal Gastroenterology, 1992.pdf*. 1992, The American Journal of Gastroenterology.

23. Heianna, J. and T. Miyauchi, *<Spontaneous regression of multiple lung metastases following regression of hepato after transcatheter arterial embolization, Hepatogastroenterology, 2007.pdf>.* Journal of Hepatogastroenterology, 2007. **54**: p. 1560-1562.

24. Harimoto, N., et al., *Spontaneous regression of multiple pulmonary recurrences of hepatocellular carcinoma after hepatectomy: Report of a case.* Surgery Today, 2012. **42**(5): p. 475-478.

25. Ayres, R.C., et al., *Spontaneous regression of hepatocellular carcinoma.* Gut, 1990. **31**(6): p. 722-4.

26. Bastawrous, S., M.J. Kogut, and P. Bhargava, *Spontaneous regression of hepatocellular carcinoma in a cirrhotic patient: Possible vascular hypothesis.* Singapore Medical Journal, 2012. **53**(10): p. 2-5.

27. Izuishi, K., M. Ryu, and T. Hasebe, *Spontaneous total necrosis of hepatocellular carcinoma, report of a case*. 2000, Hepato-Gastroenterology. p. 1122-1124.

28. Gaffet, M.J. and J.P. Joyce, *<Spontaneous regression of hepatocellular carcinoma-Gaffey.pdf>.* Cancer, 1990.

29. Van Halteren, H.K., J.M.J.I. Salemans, and H. Peters, *<Spontaneous regression of hepatocellular carcinoma-Hendrik K Van Halteren.pdf>.* Journal of Hepatology, 1997. **27**: p. 211-215.

30. Kaczynski, J., et al., *Spontaneous regression of hepatocellular carcinoma Case report.* 1998: p. 147-150.

31. Misawa, K., et al., *Case report Spontaneous regression of hepatocellular carcinoma.* Journal of Gastroenterology, 1999(October 2002): p. 410-414.

32. Uenishi, T., et al., *Spontaneous Regression of a Large Hepatocellular Carcinoma with Portal Vein Tumor Thrombi: Report of a Case.* Surg Today Jpn J Surg, 2000. **30**: p. 82-85.

33. Nakai, T., T. Shimomura, and F. Hirokawa, *Spontaneous regression of recurrent hepatocellular carcinoma after TAE: possible mechanisms of immune mediation.* International journal of clinical oncology / Japan Society of Clinical Oncology, 2001. **6**(3): p. 149-52.

34. Ohta, H., et al., *Spontaneous regression of hepatocellular carcinoma with complete necrosis: case report.* Abdom Imaging, 2005. **30**(6): p. 734-7.

35. Kondo, S., et al., *Spontaneous regression of hepatocellular carcinoma.* International Journal of Clinical Oncology, 2006. **11**(5): p. 407-411.

36. Vardhana, H.G. and M. Panda, *Spontaneous regression of hepatocellular carcinoma: potential promise for the future*. 2007. p. 223-224.

37. Yang, F., D. Fu, and Q. Ni, *Resectability of Pancreatic Cancer and Diabetes.* The American Journal of Gastroenterology, 2008. **0**(0): p. 080326032703303-???

38. Del Poggio, P., et al., *The mysterious case of spontaneous disappearance of hepatocellular carcinoma.* Digestive and Liver Disease, 2009. **41**(7): p. 21-25.

39. Iwasaki, M., et al., *Spontaneous regression of hepatocellular carcinoma: a case report.* Japanese journal of clinical oncology, 1997. **27**(4): p. 278-281.

40. Lam, K.C., J.C.I. Ho, and R.T.T. Yeung, *Spontaneous Regression of Hepa tocellular Carcinoma A Case Study.* Cancer, 1982. **50**: p. 332-336.

41. Tocci, G., et al., *Spontaneous remission of hepatocellular carcinoma after massive gastrointestinal haemorrhage Coffee consumption as trigger for insulin dependent diabetes mellitus in childhood.* 1988. **300**.

42. *<A Case of Spontaneous Regression of Hepatocellular Review of Literture.pdf>.*

43. Nishijima, N., et al., *Education and Imaging. Hepatobiliary and pancreatic: spontaneous regression of hepatocellular cancer demonstrated by contrast-enhanced ultrasonography.* J Gastroenterol Hepatol, 2009. **24**(6): p. 1153.

44. Feo, C.F., A. Marrosu, and A.M. Scanu, *Spontaneous regression of hepatocellular carcinoma: Report of a Case.* European journal of gastroenterology & hepatology, 2004(16): p. 933-936.

45. Oquiñena, S., et al., *Spontaneous regression of hepatocellular carcinoma: Three case reports and a categorized review of the literature.* Digestive Diseases and Sciences, 2009. **54**(5): p. 1147-1153.

46. Cheng, H.M. and M.C. Tsai, *Regression of hepatocellular carcinoma spontaneous or herbal medicine related?* American Journal of Chinese Medicine, 2004. **32**(4): p. 579-585.

47. Sato, Y., Y. Ohta, and P. Regression, *Case of Spontaneous Regression.* 1985(April 1980).

48. Abiru, S., et al., *Spontaneous regression of hepatocellular carcinoma associated with elevated levels of interleukin 18.* The American journal of gastroenterology, 2002. **97**(3): p. 774-775.

49. Hong, J.H. and D.D. Seo, *A case of spontaenous regression of hepatocellular carcinoma with multiple lung metastases.* Korean Journal of Gastroenterology, 2010. **55**: p. 133-138.

50. Nam, S.W., J.-Y. Han, and J.I. Kim, *Spontaneous regression of a large hepatocellular carcinoma with skull metastasis.* Journal of Gastroenterology and Hepatology, 2005. **20**: p. 488-492.

51. Stoelben, E., et al., *Spontaneous regression of hepatocellular carcinoma confirmed by surgical specimen: Report of two cases and review of the literature.* Langenbeck's Archives of Surgery, 1998. **383**(6): p. 447-452.

52. Nouso, K., et al., *Regression of hepatocellular carcinoma during vitamin K administration.* World Journal of Gastroenterology, 2005. **11**(42): p. 6722-6724.

53. Sanz, R.G., E.M. Gonzalez, and F.C. Ruiz-Delgado, *Spontaneous Regression of a Recurrent Hepatocellular Carcinoma.* Digestive Diseases and Sciences, 1998. **43**(2): p. 323-328.

54. Peddu, P., et al., *Vanishing liver tumours.* Clin Radiol, 2008. **63**(3): p. 329-39.

55. Ohtani, H., et al., *Spontaneous regression of hepatocellular carcinoma: Report of a case.* Surgery Today, 2005. **35**(12): p. 1081-1086.

56. Kato, H., et al., *Spontaneous regression of hepatocellular carcinoma: two case reports and a literature review.* Hepatol Res, 2004. **29**(3): p. 180-190.

57. Hsu, C.Y., et al., *Spontaneous regression of advanced hepatocellular carcinoma: a case report.* Cases J, 2009. **2**: p. 6251.

58. Yano, Y., F. Yamashita, and K. Kuwaki, *Partial spontaneous regression of hepatocellular carcinoma: a case with high concentrations of serum lens culinaris agglutinin-reactive alpha fetoprotein*. 2005. p. 97-103.

59. Takeura, C., T. Tokoro, and Y. Tanahashi, *Abstracts of the Twelfth Annual Americas Hepato-Pancreato-Biliary Congress. March 7-11, 2012. Miami Beach, Florida, USA.* HPB : the official journal of the International Hepato Pancreato Biliary Association, 2012. **14 Suppl 1**(March 2012): p. 1-91.

60. Arora, N. and S. Madhusudhana, *Spontaneous regression of hepatocellular cancer: Case report and review of literature.* Gastrointestinal Cancer Research, 2011. **4**(4): p. 141-143.

61. Morimoto, Y., et al., *Spontaneous necrosis of hepatocellular carcinoma: a case report.* Dig Surg, 2002. **19**(5): p. 413-8.

62. Gottfried, E.B., R. Steller, and F. Paronetto, *Spontaneous regression of hepatocellular carcinoma Gottfired.* Gastroenterology, 1982. **82**: p. 770-774.

63. Royuela, S.F., *Prolonged course of hepatocellular carcinoma A spontaneous regression.* Gastroenterlogia Y. Hepatologia, 1989. **12**(10).

64. Bruix, J., et al., *Spontaneous regression of hepatocellular carcinoma*. 1992. p. 329-333.

65. Herrera, A., J.C. Erdozain, and E. Molina, *Spontaneous regression of hepatocellular carcinoma（小松先生）.pdf*. 1996.

66. McCaughan, G.W., M.J. Bilous, and N.D. Gallagher, *Long???term survival with tumor regression in androgen???induced liver tumors.* Cancer, 1985. **56**(11): p. 2622-2626.

67. Takayasu, K., Y. Muramatsu, and Y. Shima, *Necrosis of hepatocellular carcinoma as a result of subintimal injury.pdf*. 1986.

68. Chiesara, F., et al., *A case of hepatocellular carcinoma: Spontaneous regression?* Digestive and Liver Disease, 2014. **46**(7): p. 659-660.

69. Kumar, A. and D.T. Le, *Hepatocellular carcinoma regression after cessation of immunosuppressive therapy.* Journal of Clinical Oncology, 2016. **34**(10): p. e90-e92.

70. Park, H.S., et al., *Hepatocellular carcinoma with massive lymphoid infiltration: A regressing phenomenon?* Pathology Research and Practice, 2009. **205**(9): p. 648-652.

71. Yang, S.Z., et al., *Recurrence of Hepatocellular Carcinoma With Epithelial-Mesenchymal Transition After Spontaneous Regression: A Case Report.* Medicine (Baltimore), 2015. **94**(28): p. e1062.

72. Verla-Tebit, E. and O.E. Rahma, *Regression of hepatocellular carcinoma after treatment of hepatitis C: a case report.* J Gastrointest Oncol, 2015. **6**(3): p. E52-4.

73. Mochizuki, T. and Y. Takehara, *Regression of hepatocellular carcinoma.* AJR, 1991.

74. Saito, T., et al., *Spontaneous regression of a large hepatocellular carcinoma with multiple lung metastases.* Gut Liver, 2014. **8**(5): p. 569-74.

75. Erturk, S., et al., *Spontaneous regression of hepatocellular carcinoma: a case report*. 2004. p. 95-98.

76. Yamamoto, S., et al., *Spontaneous regression of hepatocellular carcinoma after improving diabetes mellitus: possibly responsible for immune system.* Kanzo, 2012. **53**(3): p. 167-174.

77. Wang, Z., et al., *The clue of a possible etiology about spontaneous regression of hepatocellular carcinoma: a perspective on pathology.* Onco Targets Ther, 2015. **8**: p. 395-400.

78. Tsai, S.C., J.L. Kao, and C.C. Shiao, *Spontaneous regression of a hepatoma with ring calcification.* Acta Clinica Belgica, 2014. **69**(2): p. 130-131.

79. Okano, A. and M. Ohana, *Spontaneous regression of hepatocellular carcinoma: its imaging course leading to complete disappearance.* Case Rep Oncol, 2015. **8**(1): p. 94-100.

80. Okano, A., et al., *Spontaneous regression of hepatocellular carcinoma due to disruption of the feeding artery.* Case Reports in Oncology, 2013. **6**(1): p. 180-185.

81. Jozuka, H., et al., *Psycho-neuro-immunological treatment of hepatocellular carcinoma with major depression – a single case report.* Current Medical Research and Opinion, 2003. **19**(1): p. 59-63.

82. Matsuoka, S., et al., *Pathological evidence of the cause of spontaneous regression in a case of resected hepatocellular carcinoma.* Intern Med, 2015. **54**(1): p. 25-30.

83. Tomino, T., et al., *Spontaneous massive necrosis of hepatocellular carcinoma with narrowing and occlusion of the arteries and portal veins.* Case Rep Gastroenterol, 2014. **8**(1): p. 148-55.

84. Suzuki, M., et al., *Spontaneous Regression of Hepatocellular Carcinoma--a case report, Suzuki M, Okazaki, Hepatogastroenterology, 1989.pdf.* New York, 1989. **36**: p. 160-163.

85. Liai, T., et al., *Spontaneous Complete Regression of Hepatocellular Carcinoma with Portal Vein Tumor Thrombus, Hepatogastroenterology, 2003.pdf.* Hepato-Gastroenterology, 2003. **50**: p. 1628-1630.

86. Magalotli, D., C. Gueli, and M. Zoli, *Transient spontaneous regression of hepatocellular carcinoma*. 1998. p. 2369-2371.

87. Sugamoto, Y., et al., *Spontaneous regression of hepatocellular carcinoma in an obese patient.* 2015: p. 59-63.

88. Ikeda, M., et al., *Spontaneous regression of hepatocellular carcinoma with multiple lung metastases: a case report.* Jpn.J.Clin.Oncol., 2001. **31**(0368-2811 (Print)): p. 454-458.
