# Supplementary material for: Spontaneous Regression of Hepatocellular Carcinoma and Review of Reports in the Published English Literature
Source: Case Rep Med. 2019 Mar 31;2019:9756758. doi: 10.1155/2019/9756758 (PMC6462316; doi:10.1155/2019/9756758)
Supplement: Supplementary Materials — Table S1: patient's clinical history organized as a timeline. Table S2: characteristics of patients with spontaneous regression (SR) of HCC. Table S3: resected cases of HCC that regressed spontaneously, with radiographic and pathologic features that demonstrate vascular insufficiency and/or an inflammatory response. [file 9756758.f1.zip › table s3-resected cases of hcc that regressed spontaneously_CRIM_2680985.docx]

Table S3. Resected cases of HCC that regressed spontaneously, with radiographic and pathologic features that demonstrate vascular insufficiency and/or an inflammatory response.

| **Paper Citation** |  | **Size (cm)** | **Necrosis Present** | **Evidence of Vascular Accident** | **Evidence of Inflammatory Response** |
| --- | --- | --- | --- | --- | --- |
| 1. Izuishi et al. [1] | 2000 | 3.5 | Y | Tumor appeared hypovascular on CT angiogram, tumor was surrounded by an area of enhancement | Mild inflammatory infiltrate around tumour |
| 2. Markovic et al.[2] | 1996 | 13 | Y |  | Tumor contained necrosis with pseudocapsular collagenous lining infiltrated with lymphocytes. Liver parenchyma surrounding tumour necrosis reflected severe inflammatory infiltration with lymphocytes. |
| 3. Zimmerman et al.[3] | 2002 | 6 | N | Extensive invasion of intrahepatic portal vein branch, forming almost a complete vascular cast | Dense lymphocyte invasion within tumor |
| 4. McDermott and Khettry[4] | 1994 | 13 | Y | Hepatic arterial angiogram showed large hypervascular mass in left lobe, capillary phase showed nodular irregularities at the peripheral margin of the mass. Liver parenchyma surrounding the mass was normal. Small portal vein tumor thrombus in adjacent liver tissue. |  |
| 5. Imaoka et al. [5] | 1994 | 3.5 | Y | Thrombus seen in a relatively large artery in non-tumor liver tissue |  |
| 6. Nakajima et al. [6] | 2004 | 6.8 | N | Tumour had slightly high density content, and no marginal enhancement, it was hypovascular. Aspiration cytology revealed bloody fluid. |  |
| 7. Li et al. [7] | 2003 | 3 | Y | Hypovascular HCC on angiogram | Cut surface of resected lesion showed a growth of connective tissue with lymphocytes but malignant cells. Prenchyma surrounding necrotic tissue was composed of necrotic tumour |
| 8. Liai et al.[8] | 2003 | 7 | Y | Portal venous thrombus. Tumour was completely necrotic tissue, as was the tumour thrombus |  |
| 9. Ozeki et al.[9] | 1996 | 5 | Y | Hypovascular HCC on angiogram | Microscopic examination of cut surface revealed growth of connective tissue with inflammatory cell infiltration. |
| 10. Meza-Junco et al.[10] | 2007 | 6 | Y | Necrosis surrounding the tumor in the absence of linfocitary infiltration |  |
| 11. Storey et al. [11] | 2011 | 8 | Y | Tumour was hypovascular. | Chronic inflammation and hemosiderin laden macrophages. |
| 12. Matsuo et al.[12] | 2001 | 3.5 | Y | Hypovascular on angiography | The lesion showed the granulation tissue, which is composed of fibroblasts, lymphocytes, and hemosiderin laden macrophages. |
| 13.Harimoto et al.[13] | 2012 | 9 | Y | CT showed a tumor thrombus in the right hepatic vein, and a low density mass that was peripherally enhanced. Resected section of the tumour revealed a necrosed mass, hepatic vein invasion was observed. |  |
| 14.Uenishi et al.[14] | 2000 | 12 | Y | Hypovascular on angiography. Macroscopically, the tumour was hemorrhagic, and was surrounded by a thick fibrous capsule. The tumors contained only coagulative necrosis, and several areas were surrounded by inflammatory cells. The parenchyma of the liver surrounding the tumors showed chronic mild hepatitis with bridging fibrosis. |  |
| 15. Ohta et al. [15] | 2005 | 6 | Y | Cut surface shows necrotic tissue, and hemorrhage and a fibrous capsule. The entire tumour had undergone coagulation necrosis. | Some granulomatous lesions with inflammatory cell infiltration were found in the noncancerous liver tissue around the main tumor. |
| 16. Stoelben et al.[16] | 1998 | 6 | Y | No evidence of vascular accident |  |
| 17. Stoelben et al.[16] | 1998 | 5 | Y | Histologic assessment showed totally necrotic tissue with small islets of primary liver hepatocellular carcinoma. |  |
| 18. Hsu et al.q[17] | 2006 | 6 | Y |  | Inflammatory cell infiltration into fibrous capsule and inside the tumor. |
| 19. Yano et al.[18] | 2005 | 3 | Y | Possible ruptured artery mentioned as one of the causes of coagulation necrosis | Fibrous capsule thickened due to granulation tissue with infiltration of inflammatory cells and macrophages. |

**REFERENCES**

1. Izuishi, K., M. Ryu, and T. Hasebe, *Spontaneous total necrosis of hepatocellular carcinoma, report of a case*. 2000, Hepato-Gastroenterology. p. 1122-1124.

2. Markovic, S. and V. Ferlan-Marolt, *Spontaneous regression of hepatocellular carcinoma.* American Journal of Gastroenterology, 1996. **91**(2): p. 392-393.

3. Zimmermann, A., et al., *Hepatocellular carcinoma with an unusual medullary-like histology and signs of regression ("medullary-like hepatocellular carcinoma").* Dig Liver Dis, 2002. **34**(10): p. 748-753.

4. McDermott, W.V. and U. Khettry, *Clear cell carcinoma of the liver with spontaneous regression of metastases.* J Surg Oncol, 1994. **57**(3): p. 206-209.

5. Imaoka, S. and Y. Sasaki, *Necrosis of hepatocellular Carcinoma Caused by Spontaneously Arising Arterial Thrombus 1994.* Hepato-Gastroenterology, 1994. **41**: p. 359-362.

6. Nakajima, T., et al., *Recurrence of hepatocellular carcinoma with rapid growth after spontaneous regression.* World journal of gastroenterology : WJG, 2004. **10**(22): p. 3385-7.

7. Li, A.J., et al., *Spontaneous complete necrosis of hepatocellular carcinoma: A case report*. 2003. p. 152-154.

8. Liai, T., et al., *Spontaneous Complete Regression of Hepatocellular Carcinoma with Portal Vein Tumor Thrombus, Hepatogastroenterology, 2003.pdf.* Hepato-Gastroenterology, 2003. **50**: p. 1628-1630.

9. Ozeki, Y., N. Matsubara, and K.-i. Tateyama, *Spontaneous complete necrosis of hepatocellular carcinoma-Ozeki.pdf.* American Journal of Gastroenterology, 1996. **91**(2).

10. Meza-Junco, J. and A.J. Montano-Loza, *Spontaneous partial regression of hepatocellular carcinoma in a cirrhotic patient, Annals of Hepatology 2007.* Annals of Hepatology, 2007. **6**: p. 66-69.

11. Storey, R.E., et al., *Spontaneous complete regression of hepatocellular carcinoma.* Med Oncol, 2011. **28**(4): p. 948-50.

12. Matsuo, R., et al., *Spontaneous regression of hepatocellular carcinoma - A case report.* Hepato-Gastroenterology, 2001(48): p. 1740-1742.

13. Harimoto, N., et al., *Spontaneous regression of multiple pulmonary recurrences of hepatocellular carcinoma after hepatectomy: Report of a case.* Surgery Today, 2012. **42**(5): p. 475-478.

14. Uenishi, T., et al., *Spontaneous Regression of a Large Hepatocellular Carcinoma with Portal Vein Tumor Thrombi: Report of a Case.* Surg Today Jpn J Surg, 2000. **30**: p. 82-85.

15. Ohta, H., et al., *Spontaneous regression of hepatocellular carcinoma with complete necrosis: case report.* Abdom Imaging, 2005. **30**(6): p. 734-7.

16. Stoelben, E., et al., *Spontaneous regression of hepatocellular carcinoma confirmed by surgical specimen: Report of two cases and review of the literature.* Langenbeck's Archives of Surgery, 1998. **383**(6): p. 447-452.

17. Hsu, C.Y., et al., *Spontaneous regression of advanced hepatocellular carcinoma: a case report.* Cases J, 2009. **2**: p. 6251.

18. Yano, Y., F. Yamashita, and K. Kuwaki, *Partial spontaneous regression of hepatocellular carcinoma: a case with high concentrations of serum lens culinaris agglutinin-reactive alpha fetoprotein*. 2005. p. 97-103.
